# Development and experimental validation of a dynamic numerical model for human articular cartilage

**DOI:** 10.1177/09544119231180901

**Published:** 2023-06-22

**Authors:** Ben Mellors, Piers Allen, Carolina E Lavecchia, Sophie Mountcastle, Megan E Cooke, Bernard M Lawless, Sophie C Cox, Simon Jones, Daniel M Espino

**Affiliations:** 1 Physical Sciences for Health CDT, Department of Chemistry, University of Birmingham, Birmingham, UK; 2 Department of Mechanical Engineering, University of Birmingham, Birmingham, UK; 3 Institute of Inflammation and Ageing, University of Birmingham, Birmingham, UK; 4 School of Chemical Engineering, University of Birmingham, Birmingham, UK

**Keywords:** Articular cartilage, dynamic mechanical analysis, finite element analysis, hyperelastic, transient

## Abstract

The purpose of this study was to create a preliminary set of experimentally validated Finite Element Analysis (FEA) models, in order to predict the dynamic mechanical behaviour of human articular cartilage (AC). Current models consider static loading with limited independent experimental validation, while the models for this study assess dynamic loading of AC, with direct comparison and validation to physical testing. Three different FEA models of AC were constructed, which considered both linear elastic and hyperelastic models; Neo-Hookean and Ogden. Models were validated using the data collected from compression testing of human femoral heads across 0–1.7 MPa (quasi-static tests and dynamic mechanical analysis). The linear elastic model was inadequate, with a 10-fold over prediction of the displacement dynamic amplitude. The Neo-Hookean model accurately predicted the dynamic amplitude but failed to predict the initial compression of the cartilage, with a 10 times overprediction. The Ogden model provided the best results, with both the initial compression lying within one standard deviation of that observed in the validation data set, and the dynamic amplitude of the same order of magnitude. In conclusion, this study has found that the fast dynamic response of human AC is best represented by a third order Ogden model.

## Introduction

In the UK, 8.75 million people sought treatment for Osteoarthritis (OA) between 2004 and 2010,^1^ with this number expected to rise with both an increasing and ageing population. In the US, an estimated 27 million adults had one or more types of OA in 2008; an increase from the 21 million estimated in 1995.^2^ Macroscopically, healthy articular cartilage (AC) is a soft, smooth and glossy connective tissue that covers the underlying bone in synovial joints, preventing bone-bone contact. The disease affects the AC of these joints, such as the knee and hip. It involves AC degradation along with joint pain potentially from a bone-on-bone contact within the joints, as the typically smooth articulating^3^ surface becomes damaged and has been shown to reduce the elastic response of AC.^4^

AC is an inhomogeneous, anisotropic material with a complex structure adapted to withstand repeated high stress loads with little or no damage over its lifetime.^5,6^ In terms of its dynamic behaviour, its ability to store and dissipate energy has been characterised as a function of the loading frequency^7,8^; measured using a technique known as Dynamic Mechanical Analysis (DMA). AC material properties have been described by a variety of different models, including biphasic, triphasic, hyperelastic, poroelastic, viscoelastic and fibril reinforced. Although each of these material characterisations are widely accepted, contentions have been made. Kovach^9^ described cartilage as a hydrogel, a complex liquid and not a porous rock, while Huyghe et al.^10^ suggested inconsistencies with model energetics. For all models created the validation of the material properties via an experimental comparison of the mechanical behaviour is key. Often, validation is performed through the same data as used to build the model, thus reducing the validity of the model as a true and generic representation of AC.

AC in the lower limbs experiences higher stresses compared to joints found in the upper body, with estimates found in the range of 1–6 MPa, during walking^11^ and climbing up and down stairs.^12^ This loading is also dynamic, with loading components, such as the heel-strike, acting at an equivalent frequency of 3–5 Hz for the general population.^13^ However, a subset of the population with a predisposition to OA exhibit rapid heal strike rise, with a much larger frequency of dynamic loading, at approximately 90 Hz.^14,15^ Despite the known effect of physiological induced stress conditions of cartilage being known,^8^ the majority of models within the literature consider stress below this range, with stresses of 10–100 kPa.^16^ These models then deviate further from the known conditions by only considering static load conditions or loading at rates much lower than that observed physiologically during non-equilibrium dynamic states. It has previously been shown that static compression suppresses the biological activity of chondrocytes in AC specimens whereas dynamic loading either supresses or enhances the response depending on the frequency and magnitude of the cyclic load.^7,17^ Further, it has recently been demonstrated that the mechanical behaviour of cartilage differs at lower than physiological frequencies of loading^18^ and at lower than physiological loading.^8^

Accurately modelling the mechanical properties of AC is an important step in developing novel prosthetic devices or replacement biomaterials, as might be necessary during OA. Regulatory bodies, such as the U.S. Food & Drug Administration, are now encouraging the use of modelling data alongside clinical data, for new device or drug approval.^19^ Finite element analysis (FEA) can be used to create computational models of the behaviour of AC, with the aim of improving the understanding of the relationship between structure and mechanical response. FEA has been used to model AC through varying approaches, with models considering cartilage as a single material,^20^ and those based on the mathematical models such as a bi- and tri-phasic theory.^21,22^ These models can the then be subdivided into those that consider the material to be simply linear elastic,^23^ or more advanced in nature such as hyperelastic,^24,25^ viscoelastic^16^ or poroelastic.^26^ Some of these models also consider the orientation of the fibrous collagen network^27^; however, varying simplifications are made throughout all model types mentioned.

Despite the large range of material properties and models in the literature, limited work has been done to model how cartilage behaves dynamically, which describes the physiological behaviour of the tissue during its fast dynamic response to non-equilibrium conditions such as walking and beyond. Many models also lack the independent validation of their findings, with results of model predictions often compared to the data sets used to determine the material parameters for that given study. Therefore, these two factors, along with the lack of both physiological loading and frequencies applied to current models provide the motivation for this study.

The aim of this study was to create preliminary models that accurately represent the compressive behaviour of human AC during physiological loading conditions. These models consider both the dynamic amplitude, which is defined as the change in compression (displacement) across the physiological range, and the initial compression, from no induced pressure to mean physiological pressure. Physical testing of human AC is used in order to determine the material properties for each model, with a second independent human dataset tested to experimentally validate the model findings.

## Materials and methods

### Experimental testing

To generate the initial data to build each of the models, experimental testing of human AC specimens was performed. AC specimens were obtained from five human femoral heads with 16 individual samples harvested. The femoral heads were donated by patients who underwent surgery following traumatic fracture of the femoral neck. The sample harvested were divided as follows: 10 samples for model creation and six samples for validation. Ethical approval for this study was provided by the United Kingdom National Research Ethics Service (East of Scotland Research Ethics Service; 11/ES/1044) and consent for the use of their tissue for research was given by the patients.

**Figure 1.**
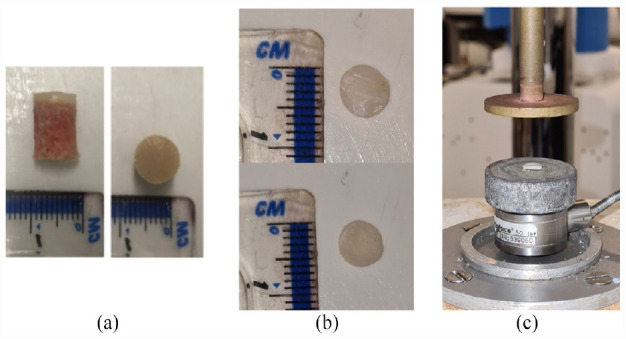
(a) Example of cartilage-bone core prior to dynamic mechanical analysis,^32^ (b) example of cartilage disc separated from the core using a medical scalpel ensuring no bone is left on sample and (c) sample in situ prior to DMA.

Each femoral head was stored at −80°C until 24 h before testing. Cartilage on-bone cores (8 mm diameter; *n* = 2(6)) were then harvested from femoral heads, before being defrosted to room temperature, in Ringer’s solution. This freeze-thaw has been shown to have no effect upon the dynamic mechanical properties of the AC.^28^ The harvesting process consisted of removing sections of the femoral head with a 300 mm hacksaw, shaping into 14 mm blocks. Each cube was scanned using Micro Computed Tomography (Micro-CT) and tested for another study,^29^ before the 8 mm core was extracted using a diamond coated drill bit, and the cartilage was removed using a medical scalpel^8,30^ as shown in Figure 1(b).

A Bose ElectroForce 3200 testing machine, controlled using WinTest 4.1 DMA software (Bose Corporation, Minnesota, USA; now, TA Instruments, New Castle, DE, USA) was used to perform DMA with a frequency sweep. The test protocol consisted of two separate loading methodologies: a quasi-static ramp compression and DMA. The ramp test ensures that the tissue is in a state of mechanical, static, equilibrium before the cyclic loading begins. A preload of 0.02 N was applied to the specimen and then, a quasi-static ramp test was performed, at a load rate of 3 N/s, to 61.6 N. Next, two preload cycles, 24 and 49 Hz for 1500 and 3000 cycles, respectively, were used to ensure a ‘dynamic steady-state’,^31^ followed by sweep tests at 1, 8, 10, 12, 29, 49, 71 and 88 Hz, which covers the range of frequencies observed both physiologically^32^ and those relevant to patho-physiological loading. These two mechanical tests provided experimental data to determine the initial ramp compression and subsequently the dynamic amplitude during cyclical loading, respectively, with a 30 min rehydration period in Ringer’s solution between tests. A separate dataset was used to validate the model, to independently verify that the models represent true physiological samples, as opposed to simply using the samples that were used to define the material constants. A further set of samples (*n* = 2(5)) were tested using the same protocols as above, with both the initial ramp compression tests and dynamic amplitude experiments repeated, with the full sample metrics shown in Table 1.

**Table 1.** Sample metrics.

Sample name	Model/validation	L/R hip	Age	Gender	Weight	Specimens
RHH214	Model	R	76	M	OW	4
RHH217	Model	R	72	F	NW	2
RHH238	Validation	L	85	F	OW	3
RHH239	Validation	R	71	M	NW	2

L: left; R: right; M: male; F: female; OW: overweight; NW: normal weight.

Weights categorised from patient BMI: 18.5 to 24.9 = NW, 25 to 29.9 = OW.

The thickness of each cartilage disk was also calculated using an established needle penetration technique, described elsewhere in detail.^15,33^ Following the DMA tests, each disk was rehydrated in Ringer’s solution before being measured using the needle technique, which has a resolution of 1 µm.

### Finite element analysis

#### Model set-up

**Figure 2.**
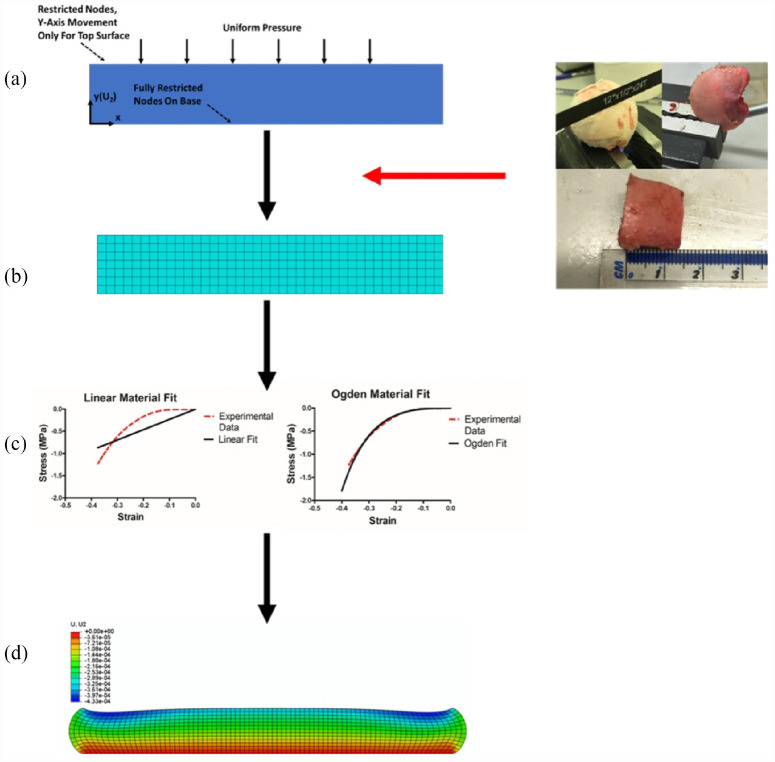
Model building flow diagram. (a) Initial set up of 2D models. (b) Geometry from physical samples used to optimise and mesh the models. (c) Linear approximation used to determine Young’s Modulus for linear models alongside ABAQUS model parameter determination for the Ogden models. Neo-Hookean parameters were taken from literature.^27^ (d) Final model under compression.

ABAQUS 6.14 (Dassault Systèmes Simulia Corp., Providence, RI, USA) software was used to model a simple two-dimensional section of cartilage. The basic geometry of all models created was a rectangle, 8 mm in length (mean), matching the cartilage cores tested, and 1.305 mm in height (mean), taken from the thickness data collected during the physical testing (Figure 2). Due to the displacement of the cartilage being negligible in the axes perpendicular to loading (*x*, and *z* in our simulation set-up), the simplification of using a cylinder and approximating this as a two-dimension model is appropriate (the loading is applied uniaxially and displacement measured along this axis too). As the material properties are to be validated, they are therefore experimentally verified for a physiological loading range allowing it to be implemented in future articular joint models. Three different models were developed; linear elastic from physical testing, Neo-Hookean hyperelastic from literature values,^24^ and Ogden hyperelastic from physical testing. Plane stress quad-elements (CPS4R) were used in all models, with mesh convergence used to determine the optimum density for each of the three models. This provided mesh sizes between 280 and 4160 elements, with element sizes of 50–200 μm (Table 2). Throughout the testing, mesh warnings were monitored for parameters including skewness (Corner angle <10° or >170°) and an aspect ratio greater than 10.

**Table 2.** Mesh convergence and element information for the three FEA models created.

Model	Number ofelements	Approximate elementsize (μm)
Linear	4160	50
Neo-Hookean	280	200
Ogden	1040	100

**Figure 3.**
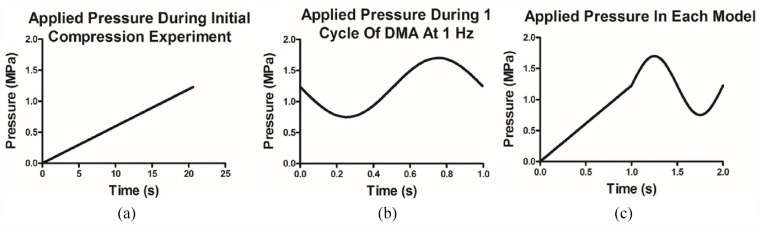
Applied pressures during experimentation and FEA models. A combination of the ramp compression physical test (a) and the 1 Hz DMA cycle (b) was applied to each model via the top surface, as a two-step process, each 1 s in duration (c).

Two different boundary conditions were applied to the model to represent the physical testing Complete restriction of both displacement and rotation was applied to the nodes at the base of the model to mimic the fixed position of the sample on the base platen as would be the case when set up on a material’s testing machine is set-up for DMA. Furthermore, to provide some further context, the model was set to mimic the test set-up; for example, a uniform pressure chosen for the FEA set-up. The cartilage samples used were always smaller that the contact platen that was applying the load, thus, the load was applied uniformly across the entirety of the surface of the tissue. The added advantage in this approach is that having the load applied uniformly allows for a direct comparison to the experimental data (or to biomaterials in future). The second restriction was applied to the top surface nodes, where they were limited to movement in the *y*-direction, which is defined as the axis perpendicular to the orientation of a plane covering the articulating AC surface, only, with both shown in Figure 3(a).

For each of the models created, uniform pressure, 0–1.7 MPa, was applied to the top surface to mimic the physical testing. This was performed with two separate steps, each 1 s in duration (Figure 3). As the material definitions of the AC models are time-independent, the 3 N/s loading during the ramp test can be reduced in time to reduce computational cost. Therefore, the ramp compression step ran for 1 s between 0 and 1.225 MPa, the mean value of the induced stress during DMA. However, subsequent sinusoidal loading was applied via a time dependant multiplication factor pressure (*a*), initial step time (*t*_0_) and current step time (*t*):

(1) $$a=1.225+0.388\cos(6.28\left(t-t_{0}\right))$$

This generates the 1 Hz sinusoidal loading experienced in the mechanical testing between 0.75 and 1.7 MPa, with the additional constant 1.225 representing the mean DMA pressure of 1.225 MPa, to generate pressure values within the range observed physiologically during ambulatory motion; that is, walking.^11^

### Characterisation of material properties for FEA

#### Linear elastic model

The simplest models created considered AC to be a linearly elastic material. Due to low physiological loading of elastic models,^23^ the Young’s modulus used for this study was determined though a linear fit to experimental stress-strain data (Figure 2(c)).

The gradient corresponds to the Young’s modulus of the material, while the Poisson’s ratio for each model was taken as 0.45.^23^ This was repeated for each of the six specimens tested, with the Young’s modulus and corresponding model used for each specimen.

#### Hyperelastic model, Neo-Hookean

The next stage was to consider the material to be hyperelastic in nature and describe its material properties through a strain energy density function. One such model,^24^ models cartilage using the Neo-Hookean strain energy density function, *W*,^34^ where:

(2) $$W=\frac{C_{10}}{2}(\bar{I_{1}}-3)$$

Where $C_{10}$ is the initial sheer modulus, and $\bar{I_{1}}$ is the first strain invariant, defined in terms of principal stretches λ_*n*_. Noting that:

(3) $$\bar{I_{1}}=\bar{\lambda_{1}}+\bar{\lambda_{2}}+\bar{\lambda_{3}}$$

Table 3 shows the different material constants, taken from literature, used for each model.

**Table 3.** Material constants used for the Neo-Hookean models created, based on literature values.

Region	Material constant $C_{10}$ (MPa)
Anterior	4.89
Posterior	5.48
Medial	5.13
Lateral	4.48

#### Hyperelastic model, Ogden

The final set of models were based on the third-order Ogden strain-energy density function. ABAQUS was used to fit different strain-energy density functions to the stress-strain data collected from the initial compression tests. Figure 2(c) shows the density function fits using Ogden for one of the specimens tested. Under the assumption of incompressibility, the Ogden model is defined as:

(4) $$W(\bar{\lambda_{1}},\bar{\lambda_{2}},\bar{\lambda_{3}})=\;\sum_{i=1}^{3}\frac{2\mu_{i}}{\alpha_{i}^{2}}\left({\bar{\lambda }}_{1}^{-\alpha_{i}}+{\bar{\lambda }}_{2}^{-\alpha_{i}}+{\bar{\lambda }}_{3}^{-\alpha_{i}}-3\right)$$

Where $\mu_{i}$ and $\alpha_{i}$ are material constants which were determined using ABAQUS, and ${\bar{\lambda }}_{i}$ are the principal stretches determined experimentally and are stated in Table 4.

**Table 4.** Material constants used for the Ogden models created, calculated from Abacus.

*i*	*µ*	*α*
1	−26,133,000	2.719
2	12,922,000	3.996
3	13,227,000	1.504

### Experimental validation

To evaluate each model created, a second set of samples were used. These samples underwent the same physical testing as the initial dataset, with both the initial compression and dynamic amplitude measured. These results were compared with the model predications. For each of the three material models used, the mean value for both the initial compression and DMA from the model predictions was validated against the mean value of the validation physical testing.

## Results

For all of the preliminary models created using the material definitions described previously, a total model running time of 2 s was employed, with a ramp step for 1 s during the initial compression up to 1.225 MPa, and a cyclic loading step, where sinusoidal loading at a frequency of 1 Hz is applied between 0.75 and 1.7 MPa. Based upon the boundary conditions applied, the displacement of the central node on the top surface was measured to determine the compression for each step within the model.

**Figure 4.**
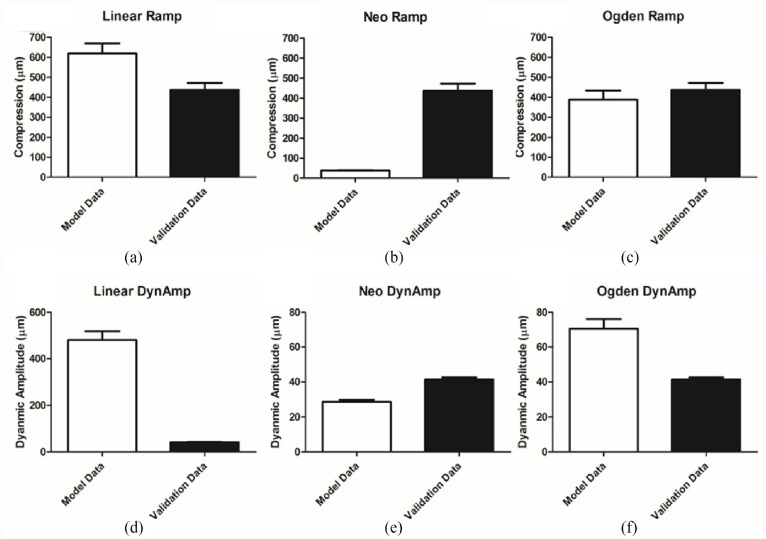
FEA comparative results panel. Each of the models generated was compared to an independent data set. Results are displayed for ramp compression on linear (a), Neo-Hookean (b), and Ogden (c) models and for dynamic amplitude compression on the same models respectively (d, e, and f). Dynamic Amplitude is shorted to DynAmp for convenience (mean ± SD).

A summary of the results is shown in Figure 4, with each of the three different model types compared to the independent validation dataset collected for both the initial compression and the dynamic amplitude. The linear model is unsuitable at predicting both aspects of the compression (Figure 4(a) and (c)). While the initial ramp compression, 618 ± 124 µm is of the same order of magnitude as the validation data, 437 ± 78 µm, the dynamic amplitude is 10× over-predicted by the linear model (480 ± 96 µm cf. a mean dynamic amplitude of 41.4 ± 2.6 µm measured experimentally).

The neo-Hookean models, with material properties based on the values determined by Henak et al.,^24^ were the first of the two models to consider the material as hyperelastic. This definition improved the modelling of the dynamic amplitude, with a mean value of 28.6 ± 2.2 µm, the same order of magnitude as that observed in the validation data set (41.4 ± 2.6 µm). However, when the performance of the ramp compression element of the model is consider, the Neo-Hookean models under predict this value by a factor of 10 at 38.6 ± 3.2 µm, when compared the independent validation data set (437 ± 78 µm).

The second of the hyperelastic models, were based on a third-order Ogden strain-energy density function. This offered the best result for the ramp compression of the three material definitions test, with a mean ramp compression modelled at 388 ± 80 µm, therefore lying within one standard deviation of the observed validation data-set value, 437 ± 78 µm. The value for the dynamic amplitude, 70.7 ± 9.5 µm, does not lie within the same accuracy, however, it is of the same order of magnitude (41.4 ± 2.6 µm). It must also be noted that of the six samples analysed for material properties using ABAQUS, only three samples showed stability under compression for the full physiological range utilised within these models.

## Discussion

This study has, for the first time, evaluated preliminary FEA models of AC utilising different material approximations that consider the same tissue deformation under a physiological stress range of 0–1.7 MPa.^11^ Using this modelling approach has the potential in the early stage assessment of artificial biomaterials. Three localised cartilage models were developed and validated utilising different material approximations all considering the same tissue deformation under a physiological stress range of 0–1.7 MPa.^11^

**Figure 5.**
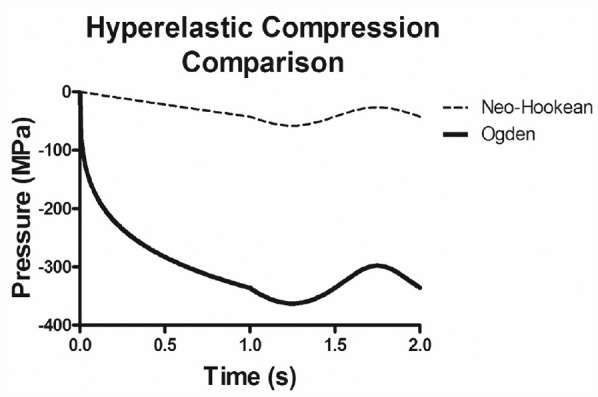
The overall compression of both the Neo-Hookean and Ogden models. This figure illustrates that although the dynamic amplitudes for both models are similar to the physical testing, the initial compression is distinctly non-linear for the Ogden model only, offering a physiological comparison.

The most representative results for modelling in the physiological loading range were observed by the third-order Ogden hyperelastic strain-energy density function. In both the ramp and the dynamic loading portions of the FEA simulation, the compressions and dynamic amplitude outputs respectively were much more comparable to the validation data set utilised here (Figure 4(c) and (f)). The ramp test was within one standard deviation of the verification set and the dynamic portion was of the same order of magnitude (437 ± 78 µm), however, it still overestimated it by approximately 58%. Compared to the Neo-Hookean models linear ramp response, this is a positive result as a non-linear output is evident in both ramp and dynamic displacement tests (Figure 5). However, Ogden has previously been reported to be inaccurate at strain rate of (0.1 and 0.025 s^−1^) when using bovine samples.^35^ This inaccuracy can be assumed in our human tissue study due to the comparability of bovine and human tissues demonstrated by multiple studies.^15,36^ Therefore, this demonstrates an instability as a complete model.

The developed linear model utilised a Young’s modulus determined from the gradient of the stress-strain curves observed in initial compression testing (Figure 3(a)). Linear approximations have been used previously, however, utilising this material assumption limits the comparability of the study as it ignores the reported biphasic properties of cartilage.^37,38^ It has been demonstrated that a linear approximation fails to capture the curvature seen in the validation tests of the AC at physiological stress backing up the concerns that linear material representations are unsuitable for this model (Figure 2(c)). The lack of ability to handle dynamic loading with the model was also observed (Figure 4(d)) with the model overestimating the validation data by over 10 times the validation data value. This led to the reasoning to include the next level of complexity, a hyperelastic material approximation.

The other hyperelastic model considered, neo-Hookean, exhibits a non-linear stress-strain relationship. The material parameters for this were adopted from work looking at patient specific loading under physiological stress of femoral heads.^24^Figure 4(b) and (e) reports the observed ramp and dynamic amplitude FEA testing results respectively in the created models. When each are considered individually in this way the dynamic test is seen to be in the same order of magnitude as an independent data set. However, the ramp test exhibits 10 times under prediction and is shown to have near linear relationship, see Figure 5. Similar to Ogden, Neo-Hookean models were also shown to be an ineffective solution at similarly low loads.^35^ This evaluation now also shows their ineffectiveness at physiological loads; thus, demonstrating the inadequacies of this model for an AC material approximation.

**Figure 6.**
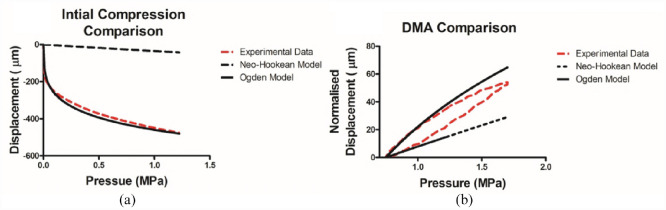
Hyperelastic model and experimental data comparisons: (a) pressure-displacement comparison, illustrating the non-linearity of the Ogden model, compared to the Neo-Hookean model and (b) hysteresis loops for the same models, demonstration the lack of time-dependency within the hyperelastic models.

To explain why the Ogden model performs better during the ramp portion and the Neo-Hookean during the dynamic portion respectively, the biological components of cartilage itself must be considered. During the ramp test, the non-linearity of cartilage is more identifiable, most likely due to going from an unloaded to loaded state. This compression, or work done, results in an increase of the entropy of the tissue due to the collagen fibre re-orientating themselves. This is part of the biological factor produces the non-linear material behaviour seen.^39,40^ This fibre characteristic is challenging to mimic with a neo-Hookean approximation whereas the Ogden approximation performs well, as demonstrated by displacement versus pressure plots of the ramp loading sequence (Figure 6(a)). The translation from pressure applied to displacement is not replicated in the Neo-Hookean approximation due to it using an advanced linear approximation. The change in crimp is less extensive during the dynamic loading as work done is used in stretching the collagen fibres/fibrils themselves, ready for recoil; thus, the closer similarity between both material models and the experimental data, as seen in Figure 6(b). Here, the normalised compression is calculated for each data point as the difference between the displacement at the minimum applied pressure during the full DMA cycle and the displacement at given data point.

Despite the comparable results of the Ogden approximation, errors are still present which show that they are not capturing the true nature of the material structure.^21,24^ The hysteresis observed in the experimental data, taken from the validation data set, in Figure 6(b) is evidence of a time-dependant variable within the compression stages, which is not addressed by any of models within this study. However, it is notable that this preliminary set of models begins to address the non-linear nature of cartilage compression in the dynamic physiological loading range; this is a novel contribution to the existing literature. There has been similar success with validation of models by DiSilvestro and Suh^41^ however, in our study we prioritised the development of an independent dataset of human samples for validation improving the validity of the results found, when compared to those that test and validate from the same data source. Material approximations that were excluded from this study were poroelastic^42,43^ and fibre reinforced models.^27,44^ Inclusion of sheer loading factor would also provide another informative variable in a future similar study as there is limited data in literature currently.^45^ The introduction of more complex models could provide better representations, however, when more parameter, or degrees of freedom, are included the accuracy of the optimisation is not always improved.^46^ Recent studies have investigated frictional torque using rotation, the techniques used could potentially be amended for measuring shear properties.

A limitation of our work is that we have not evaluated ionic transfer within the proposed material models. It has been shown by Klika et al.^47^ to affect both the initial loading and dynamic response of the tissue, however, a recent study by Aspden and Cederlund^48^ notes some of the gaps in knowledge in biphasic theory when representing non-equilibrium states such as walking or running, or other such physiological loading conditions where a loading frequency might be appropriate. An initial such study found that even at a quasi-dehydrated state of ∼0% water content, dissipative effects still took place within articular cartilage under frequency-domain loading; further, the ability to store energy was also sensitive to water content.^49^ There is a lack of currently tested biphasic model’s that have been tested under dynamic physiological loading conditions. One such study is that by Suh et al.,^7^ however, the study did not account for hyper-elastic behaviour; which has been demonstrated in this study to be relevant when representing the mechanical behaviour of human articular cartilage under dynamic loading conditions. In our current study, therefore, we have gone back a step and assumed no knowledge of the constituent parts of AC evaluating the mechanical properties of the tissue. This was in line with the aim of the study being to evaluate the limitations of material models focusing initially on the deformation response. Clearly, ionic transfer is a further step necessary in developing this model for the behaviour of cartilages dynamics. Therefore, this study addressed the non-energy dissipation component for articular cartilage with the rationale of the potential to evaluate future replacement materials using the using material parameters that were gained that naturally mimic the biological behaviour of AC.

Previously, biphasic materials have been investigated to better mimic cartilage during replacement with synthetic materials.^50^ However, the dynamic mechanical behaviour has not been addressed in terms of material replacement. Therefore, this study is a first step towards enhancing the clinical applicability of these models they could be extended to include a bone layer.^30,51^ Our focus was on biomaterials which can mimic the dynamic mechanical behaviour of natural cartilage where their material characteristics are matched in the frequency domain with possibilities being polylactic acid (PLA) polyvinyl alcohol (PVA),^52^ additive manufacture^53^ or hydrogels. The first step that was required was to mimic AC’s hyperelastic behaviour under natural loading conditions to establish bulk properties of the tissue, this is information currently available models were not able to provide. Utilising FEA models to evaluate prosthetic and implant design is well researched area with the possibility of huge translational impact. Further work to investigate how cartilage on cartilage loading impact the deformations on both sides of the joint would be informative for this goal. This, however, is limited due to the difficulties in obtaining said material from the same joint and the quality of the received tissue. Future inclusion of patient characteristics such as age, gender and anatomical region as variables would allow creation of situational specific models. This, alongside the consideration of how various biomaterials intended for cartilage replacement dissipate loading across the bone surface interface would both significantly help to aid translation.^29^ The aim of the paper was to generate and validate generic material models rather than custom to an individual, this would introduce data issues as it is currently primarily currently gathered ex vivo.

## Conclusions

Within this study, we have created and developed a series of cartilage models to test the compression during dynamic physiological loading. The simplest model, which considered the material to be linear in nature was unable to accurately replicate both the initial compression and dynamic amplitude observed in the validation data set. The first of two hyperelastic material definitions, Neo-Hookean, offered an improvement, however the best results were observed with the third-order Ogden strain-energy density function. This accurately modelled the initial compression and provided a result within the same order of magnitude for the dynamic amplitude. These models provide a preliminary assessment of cartilage modelling, with the addition of an independent validation set to improve the overall validity of the model’s outcomes. This method of evaluating and validating models within the physiologically relevant range will be very useful for future cartilage models to ensure accuracy in tissue response.
